# A cross-sectional study on the impact of the prevention and control response of the COVID-19 pandemic on minor’s orthopedic trauma in Shanghai

**DOI:** 10.1186/s13690-021-00672-7

**Published:** 2021-08-17

**Authors:** Chuang Qian, Yiming Zheng, Junrong Meng, Hao Li, Dahui Wang

**Affiliations:** 1 Department of Orthopedics, Children’s Hospital of Fudan University & National Children’s Medical Center, No. 399, Wanyuan Road, Minhang District, Shanghai, 201102 China; 2 Department of Neurosurgery, Children’s Hospital of Fudan University & National Children’s Medical Center, No. 399, Wanyuan Road, Minhang District, Shanghai, 201102 China

**Keywords:** COVID-19 pandemic, Prevention and control response level, Orthopedic trauma

## Abstract

**Background:**

The Chinese government has taken strong prevention and control measures against the COVID-19 pandemic. Although the pandemic is far from over, it has been effectively controlled in China. The outbreak of COVID-19 pandemic provides an opportunity to study the influence of governmental prevention and control response on orthopedic trauma in minors.

**Methods:**

We collected and reviewed data and information on minor’s orthopedic trauma from 1 January to 30 June of the past three year (2018, 2019 and 2020). The data were divided according to the time of prevention and control response level in 2020 (the first level response is from January 24 to March 22, the second level response is from March 23 to May 7, and the third level response is May 8 to now). By comparing the relevant data from orthopedic emergency and operating rooms from the past three years, the influence of governmental pandemic prevention measures on orthopedic trauma in minors was analyzed.

**Results:**

A total of 36,301 minors were included in the study cohort. Before the prevention and control response, the data of the orthopedic emergency department of National Children Medical Center (Shanghai) in 2020 was the same as the previous two years. The condition of children undergoing surgery at the time of injury is significantly different under different levels of prevention and control response. Under the first-level response, the number of fractures, open injuries, radial head subluxation, and surgery were significantly reduced, and the severity of patients with surgery was also significantly reduced. Under the second-level response, the number of operations began to increase, and the severity of the disease also began to rise. Under the third-level response control, the number of fractures, open injuries, and operations have returned to the levels of the previous two years. The severity of the operation has also returned to its previous level. The number of subluxations of the radial head is still different from before.

**Conclusion:**

The prevention and control response for the pandemic of COVID-19 can reduce the incidence of orthopedic trauma in minors by strengthening the guardian’s care and restricting children’s outdoor activities. With the control of the pandemic, the amount of orthopedic trauma in minors will not be affected by low-level prevention and control.

## Background

About a quarter of all minors go to the emergency department every year due to accidental injuries [1]. Fractures are the most common type of trauma in minors [2], and about a third of them will suffer from a fracture before they reach adulthood [3]. The daily incidence of fractures in China is about 3.4/1000 per year. And the incidence of fractures under 14 years old is about 1.4/1000 per year [4]. The type and severity of fractures are related to gender, region, country, weather, and culture [5, 6].

Due to the outbreak of COVID-19 pandemics in the world, the Shanghai government launched first-level prevention and control response in January 24, 2020. With the change of the pandemic situation, the response measures are constantly changing (Table 1). At present, there is no study on the impact of infectious disease prevention and control response on pediatric orthopedic trauma, and this pandemic provides the best opportunity for related research.

**Table 1:** Specific measures for prevention and control response

	First level response	Second level response	Third level response
Hospital	●Strengthen protective measures ●Strict epidemiological investigation and temperature monitoring before treatment ●COVID-19 designated hospital ●Increase doctors for Department for High Fever ●Full implementation of outpatient appointment system	●no change	● on-site registration permission
Airport and railway station	●temperature monitoring and epidemiological surveys on people coming to Shanghai ●Transfer fever patients to designated hospitals	●no change	●no change
People who come to Shanghai	●Home quarantine for people coming to Shanghai ●Suspend the requirements for people coming to Shanghai for non-work reasons ●Prohibit non- inhabitant from entering the community	●Home quarantine for people coming to Shanghai in key areas ●Appropriately Open Community ●centralized quarantine for immigrants	●Nucleic acid testing for all immigrants
Public activity	●Stop activities that can cause multitudes flocked (including tourist attractions, competitions, performances, collective religious activities, etc.)	●Appropriately open outdoor public place ●Reduce the amount of people in indoor public place to 30%	●Orderly open indoor public place
public transport	●Implement traffic sterilize ●Increase the frequency of public transportation to the minimum interval ●Prohibit the use of air conditioners	●no change	●Implement temperature monitoring in subway stations
workplace	●Extend the Spring Festival holiday by 1 week ●Implement flexible working time ●Working Online	●Resumption of Work and production under the premise of sterilize, temperature monitoring and epidemiological surveys. ●encourage working online	●Full resumption of work and production ●Nucleic acid testing for all employees
School	●Suspension of the class of all grades	●Suspension of kindergarten, Primary and secondary schools ●Graduation class restarts	●Orderly re-opening of primary and secondary schools and kindergartens ●Implement flexible lunch time

With the reduction of the response level, we can assess whether the life of the residents in Shanghai has recovered to the situation before the pandemic from the aspects of economy, life and politics. Orthopedic trauma in minors is closely related to their guardians and teachers (including kindergarten, elementary school, middle school and high school). During the pandemic, their guardianship period for minors has changed. The suspension of school reduced the supervision of teachers. The parents from dual-income families can share more time with their children during the flexible working time. So, the changes in minor’s orthopedic trauma caused by the prevention and control response can reflect the impact of the response on the lives of citizens. By analyzing the changes of pediatric orthopedic emergency department in 2020, we can understand the recovery status of the residents in Shanghai and provide some evidence for other governments to formulate relevant pandemic prevention policies.

## Methods

This study reviewed data and information of orthopedic emergency patients (less than 18 years old) at our hospital from 1 January to 30 June of the past three year (2018, 2019 and 2020). The number of emergencies, type of injuries, emergency surgeries, surgical sites, the cause of the injuries, and the severity of the injuries over the last three years were documented. For later pairing comparison of the last three years, patient information from February 29, 2020 was excluded.

### Categories of injury in the emergency room

The diagnoses of emergency patients were categorized as fractures, lacerations, contusions (including non-fracture and non-open injury), and radial head subluxations.

### Diagnostic classification of surgical patients

Based on the type of injury, we divided the diagnoses of children who required surgical intervention into open fractures, open injuries without fracture and closed fractures. The fractures are divided by surgical site: spinal fractures, clavicle fractures, pelvic fractures, metacarpals and phalanges fractures, radius and ulna fractures, humerus fractures, femur fractures, tibia and fibula fractures, and fractures of ossametatarsalia and phalanx.

### Severity of injury

**Fig. 1:**
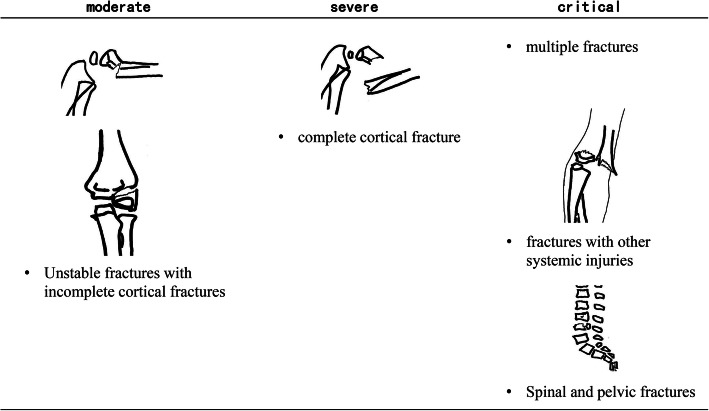
Definition of the injury severity

In order to distinguish the severity of injuries in surgical patients, we further divided the condition of all patients into three levels: moderate, severe, and critical (Fig. 1). Unstable fractures with incomplete cortical fractures were defined as moderate injuries (including type I humeral lateral condyle fractures, angled phalanx fractures, type II supracondylar fractures of the humerus, and epiphyseal fractures after reduction). A complete cortical fracture of a single limb was defined as a severe injury (including type III humeral supracondylar fractures, radial and ulnar fractures, and fractures of the tibia and fibula). Spinal and pelvic fractures, multiple fractures, or fractures with other systemic injuries were identified as critical injuries (including lumbar burst fractures, pelvic fractures, and supracondylar fractures of the humerus combined with an injury of the median nerve).

### Condition at the time of injury

We collected the information on caregiver (guardian or non-guardian) and location of injury (indoor or non-indoor) of the surgical patient at the time of injury after the prevention and control response was initiated.

### Mechanism of injury

In this study, we collected the data of minors who had been operated on due to traffic accident injuries, injury caused by falling from a height, and open injuries that occurred in the past three years; we then compared the causes.

### Unified timeline

Due to different spring festival holidays and school opening dates in the past three years, we rearranged the data for the three years using the Chinese New Year as a reference point. According to the level of response in 2020, the data of three years are divided into four groups ‘before response’, ‘first-level response’, ‘second-level response’ and ‘third-level response’. After rearrangement, the date segments of each group in the past three years are shown in the table (Table 2).

**Table 2:** The time line after unification

	2018	2019	2020
Before the response	01/21–02/14	01/12–02/03	01/01–01/23
First-level response	02/15–04/13	02.04–04/02	01/24–03/22
Second-level response	04/14–05/29	04/03–05/18	03/23–05/07
Third-level response	05/30–06/30	05/19–06/30	05/08–06/30

### Statistical method

IBM SPSS Statistics for Windows, Version 21.0 (IBM Corp., Armonk, NY, USA), was used for statistical analysis. Relevant data from the emergency room (total number of daily emergencies, fractures, open injuries, radial head subluxations) and volume of daily operations were analyzed by multiple samples pairwise comparison analyses of variance, and a sample variance homogeneity test was conducted. Age and severity of injuries in surgical patients were compared using a rank-sum test with multiple samples; the statistically significant results were further compared. A chi-square test was used to analyze the differences condition at the time of injury. A chi-square test was used to analyze the differences in causes of injury (open injury, falling from a height, and traffic accidents) in surgical patients over the past three years. *P* < 0.05 was considered statistically significant.

## Results

### General data

A total of 36,301 minors were included in this study. There were 7093 cases of fracture, 16,963 cases of contusions, 7019 cases of radial head subluxation, and 5226 cases of lacerations. Amount these cases, 1793 minors underwent surgeries, yielding a surgery rate of 4.93%.

### Emergency room data

**Fig. 2:**
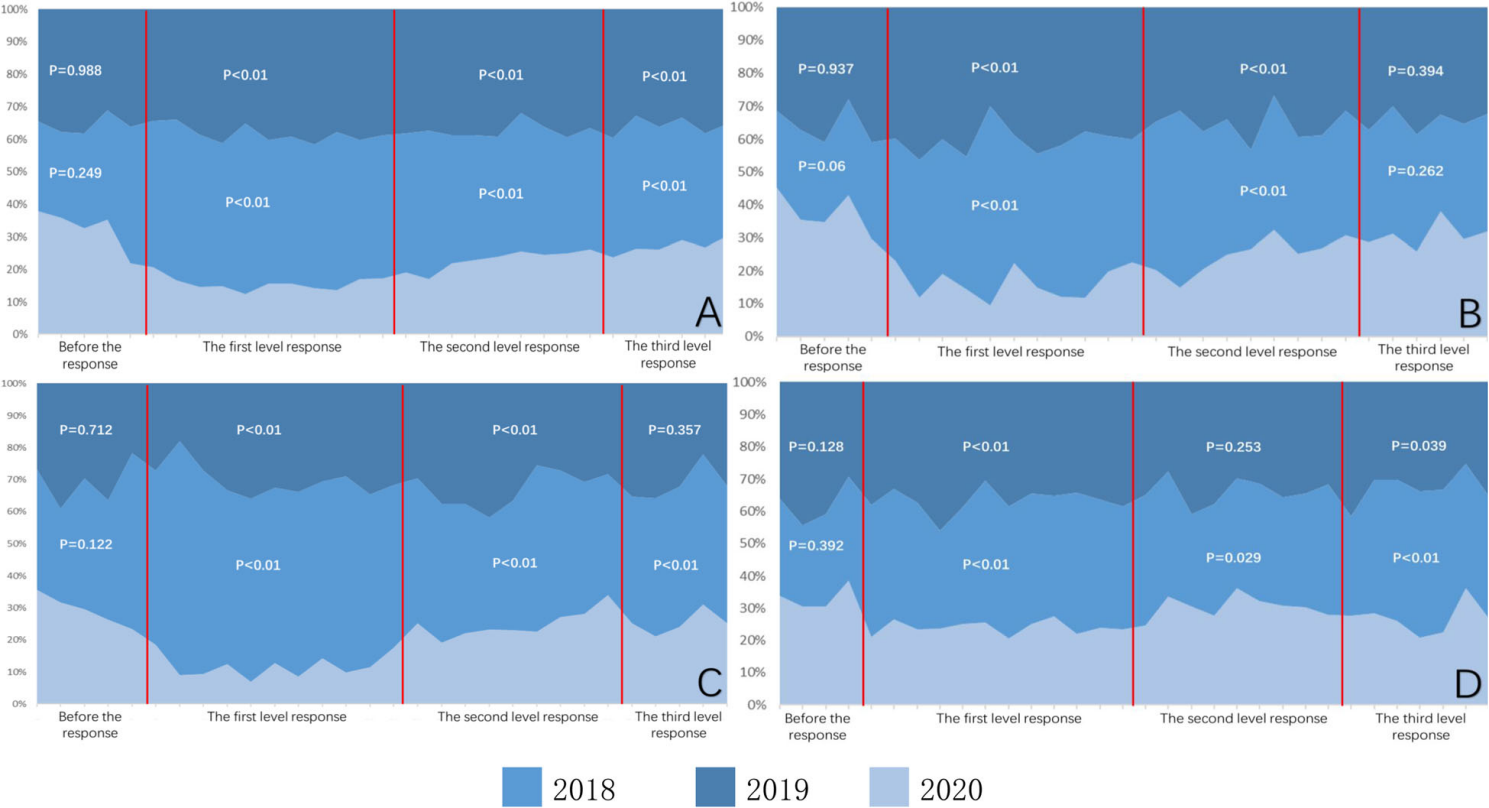
(A) Comparison of the total number of emergency cases in the last 3 years; (B) comparison of the total number of fractures in the last 3 years; (C) comparison of open injuries in the last 3 years; and (D) comparison of the number of radial head subluxations in the last 3 years

There were 14,866 emergency cases in 2018, 13,944 in 2019, and 7491 in 2020. There were statistical differences in the number of emergency cases between 2020 and the previous two years (*P* < 0.01), but no significant difference between 2018 and 2019 (*P* = 0.22). The detailed data of the emergency room visits for three years are shown in Fig. 2.

There was no significant difference between the total number of emergencies among the 3 years before the response (*P* = 0.249 and *P* = 0.988). During the response, included first-level, second-level response, and third-level response, there were significant differences in the total number of emergency treatments in 2020 compared with the previous 2 years (*P* < 0.01).

Before the response, there were no significant differences in the number of emergency fractures in 2020 compared with the previous 2 years (*P* = 0.06, *P* = 0.937). During first-level and second-level response, the number of emergency fractures in 2020 decreased significantly compared with those in the previous 2 years (*P* < 0.01). Until third-level response started, the number of emergency fractures began to rise. There was no significant difference between the number of emergency fractures in 2020 and that of the previous 2 years (*P* = 0.262 and *P* = 0.394).

Before the response, there was no significant difference in the number of lacerations between 2020 and those of the previous 2 years (*P* = 0.122 and *P* = 0.712). During the first-level and second-level response, the number of lacerations in 2020 decreased significantly compared with those in the previous 2 years (*P* < 0.01). During the third-level response, the number of lacerations in 2020 was still significantly lower than that of 2018 (P < 0.01), but there was no statistical difference with that of 2019 (*P* = 0.357).

Before the response, there was no significant difference between the number of radial head subluxations in 2020 and the previous two years (*P* = 0.392 and *P* = 0.128). In response to all levels of prevention and control, the number of radial head subluxation in 2020 decreased compared with the previous 2 years.

### Operating room data

**Fig. 3:**
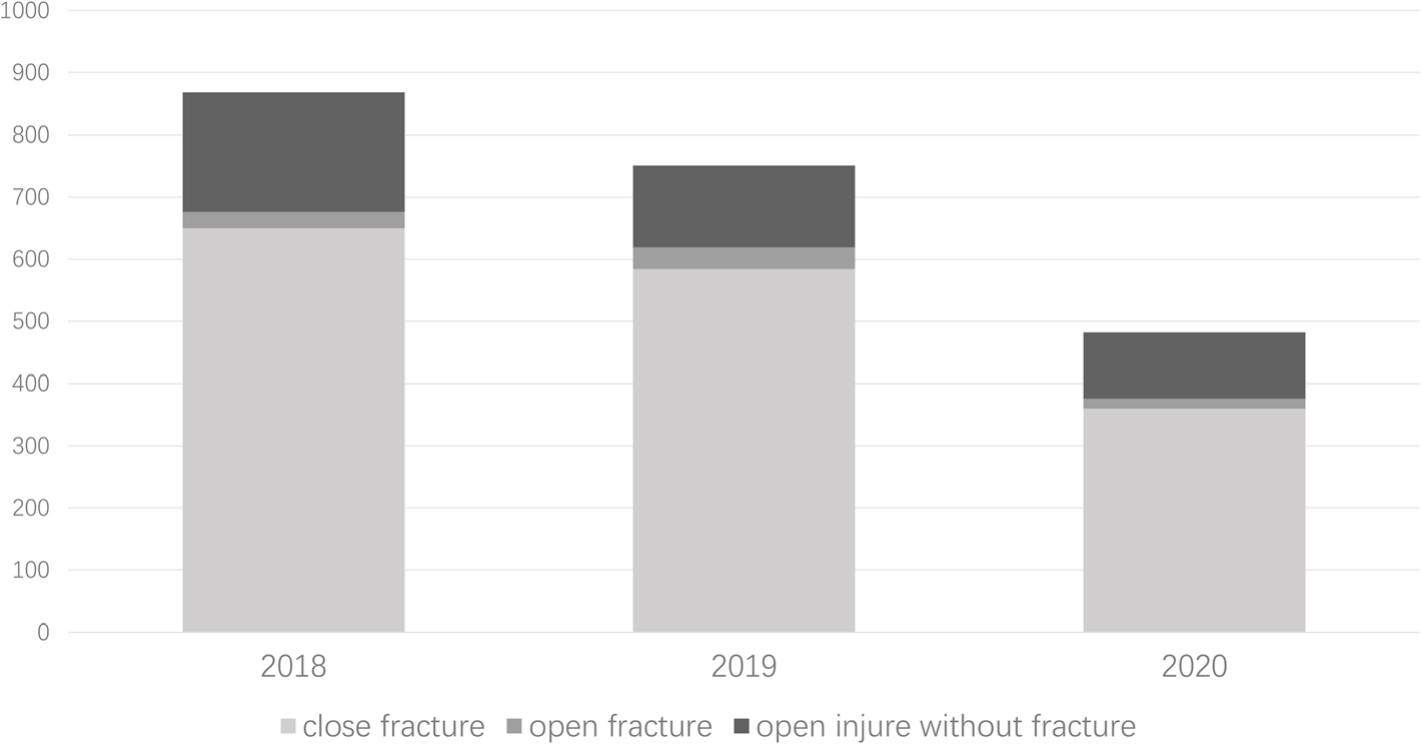
Classification of the injuries needed operation in the last 3 years

**Fig. 4:**
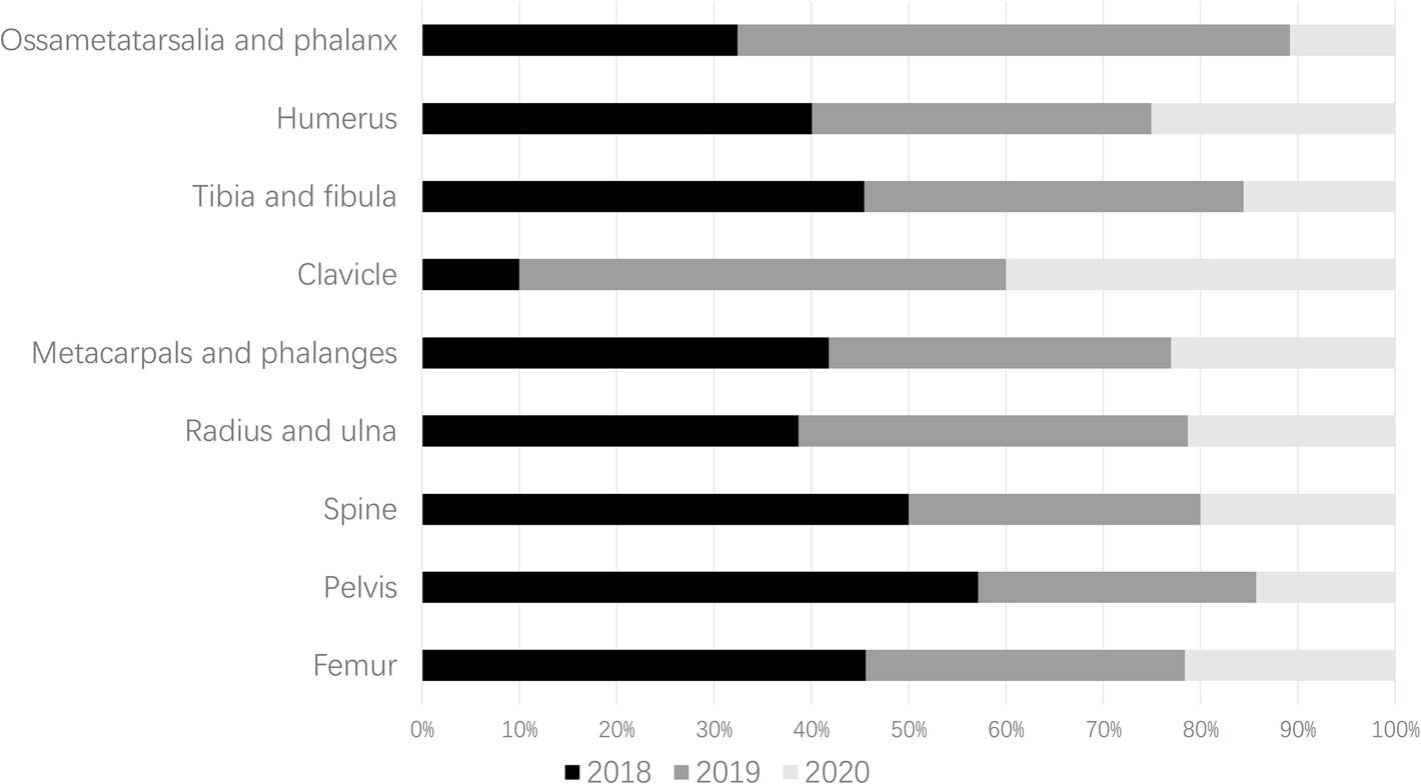
The changes in the fracture site in the last 3 years

In the past 3 years, our department has carried out a total of 1793 orthopedic emergency operating procedures, including 671 in 2018 (4.22 sets/day), 615 in 2019 (3.62 sets/day), and 507 (2.85 sets/day) in 2020; there were significant differences between the 3 years (*P* < 0.01). The difference in operations between the 3 years is shown in Figs. 3, 4.

**Fig. 5:**
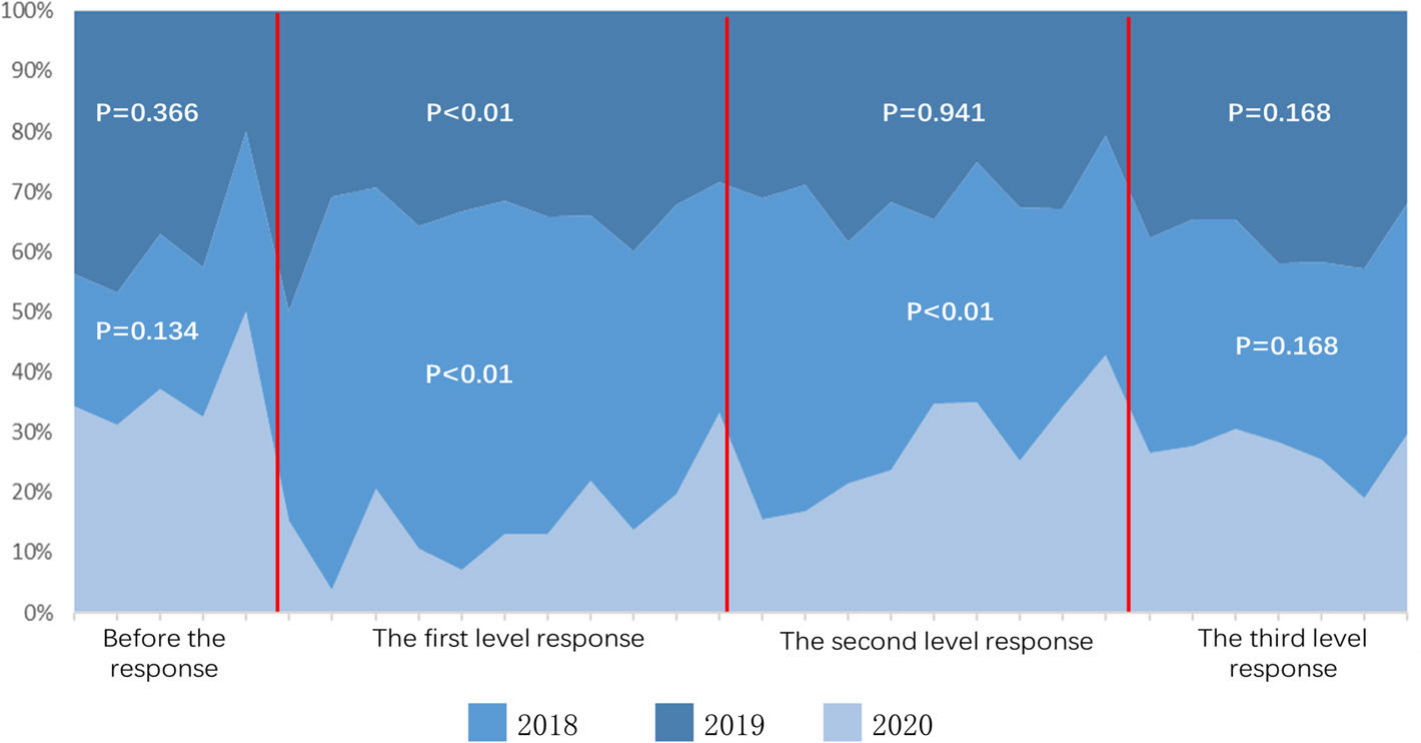
Summary of changes in the total number of emergency operations in the last 3 years

Before the response, there was no significant difference in the number of operations between 2020 and the previous 2 years (*P* = 0.134 and *P* = 0.366). Under the first-level response, the number of operations in 2020 decreased significantly (*P* < 0.01); under the second-level response, the number of operations in 2020 was significantly different from that in 2018 (P < 0.01), but there was no statistical difference between 2020 and 2019 (*P* = 0.941). At the third-level response, the number of operations recovered in 2020. There was no significant difference compared with the previous 2 years (*P* = 0.233 and *P* = 0.168) (Fig. 5).

After the prevention and control response started, the caregivers of children were significantly different under different response levels (*P* = 0.011). As the response level changed, the proportion of non-parental caregivers of injured children began to rise. Similarly, under different response levels, the location of the injured child was significantly different (*P* = 0.005). As the response level changed, the number of children injured outdoors began to rise (Table 3).

**Table 3:** Comparison of the condition at the time of injury

characteristic of inpatients	Prevention and control response level			
	First-level response	Second-level response	Third-level response	*P*-Value
Direct Parents’ care				
Yes	31 (40.4%)	80 (49.7%)	74 (38.5%)	P = 0.011
No	21 (59.6%)	81 (50.3%)	118 (61.5%)	
Indoor injury				
Yes	37 (71.2%)	89 (55.3%)	103 (53.6%)	P = 0.005
No	15 (28.8%)	72 (44.7%)	89 (46.4%)	

There was no significant difference in the severity of surgical patient between 2020 and that of the previous 2 years before the response, under the second-level response and the third-level response (*P* = 0.736, *P* = 0.528, and *P* = 0.334). During the first-level response, the severity of surgical patients in 2020 decreased significantly compared with that in the previous 2 years (*P* = 0.001; Tables 4, 5, 6 and 7).

**Table 4:** Comparison of the severity of surgical patients before the response

Year	moderate	severe	critical
2018	6	25	8
2019	8	36	19
2020	4	36	12

Overall comparison: P = 0.736

**Table 5:** Comparison of the severity of surgical patients under the first level response

Year	moderate	severe	critical
2018	28	153	49
2019	25	94	42
2020	29	35	12
Overall comparison		P = 0.001	
2020 VS 2019		*P* = 0.002	
2020 VS 2018		P < 0.01	
2019 VS 2018		*P* = 0.6

**Table 6:** Comparison of the severity of surgical patients under second-level response

Year	moderate	severe	critical
2018	34	152	60
2019	26	122	34
2020	21	108	32
Overall comparison		P = 0.528

**Table 7:** Comparison of the severity of surgical patients under third-level response

Year	moderate	severe	critical
2018	24	85	43
2019	35	124	48
2020	20	122	50

Overall comparison: P = 0.334

For the years of 2018, 2019, and 2020, 25, 19, and 10 cases of injuries by falling from a height were treated in our center, respectively, and there were significant differences between them (*P* = 0.016). The number of patients undergoing surgery due to open injuries was 218, 167, and 122, respectively. The number of patients who underwent surgery for motor vehicle accidents was 60, 53, and 30, respectively. There was no significant difference in the number of open injuries and traffic accidents between the 3 years (*P* = 0.78 and *P* = 0.563; Table 8).

**Table 8:** Comparison of the causes of injurie

	Vehicle accident	Fall from a height	Open injury
2018	60	25	218
2019	53	19	167
2020	30	10	122
*P* Value	0.563	0.016	0.78

## Discussion

As a serious global public health emergency, the impact of the COVID-19 on people’s lives all over the world has greatly exceeded that of H1N1 in 1918, H2N2 in 1957, H1N1pdm09 virus in 2009, Middle East respiratory syndrome (MERS) in 2015 and SARS in 2003. COVID-19 has a long duration and a wide range of spread, which is far beyond the previous global public health events in recent years. Under the strong control by the Chinese government, the pandemic of COVID-19 did not break out in Shanghai. On January 24, 2020, the Shanghai government announced to launch the first-level response of prevention and control. Subsequently, residents in Shanghai have experienced the whole process from the first-level response to the third-level response (Shanghai still hasn’t stopped the third-level response of prevention and control). In the process of fighting the pandemic, the economy, life, and politics have been changed with the changes of the pandemic situation. The hospital’s treatment process has also been optimized [7]. In this study, we found that minors’ orthopedic emergency also changed with the pandemic situation.

As we all know, during the Chinese New Year, the population of China’s first-tier cities will experience a tidal wave. Take Shanghai as an example. The non-Shanghai-born population, which accounts for nearly one-third of the total resident population in Shanghai, will leave Shanghai with their family members and return to their hometown for the New Year. They will return to Shanghai for work after the Spring Festival holiday. The holiday usually lasts 1 week to 2 weeks. Since the date of the Spring Festival differs every year, if the timeline is not unified by the Spring Festival, it will cause the paired data to be unable to compare in this research. After unified, we can find that there is a significant reduction in pediatric orthopedic trauma during the Spring Festival.

The only records of injury mechanisms in our study are high falls and car accidents. We have to use the data of emergency room and operating room to infer whether the injury mechanism will change due to the prevention and control response.

Before the response, the data about minors’ orthopedic trauma in Shanghai kept at the same level as the previous two years in terms of emergency number, emergency diseases, number of operations, severity of surgery, and injury mechanism. The disease spectrum did not change significantly in different years. With the change of climate and semester, the number of orthopedic trauma in minors increased with the rise of temperature and the beginning of school. Therefore, we believe that the mechanism of orthopedic trauma for minors in Shanghai have not changed in the past three years.

Through the data analysis of the injured state of children undergoing surgery in response to the pandemic, it is not difficult to find out that high-level prevention and control responses can increase parents’ direct care of children and restrict their outdoor activities. This is similar to the situation in other countries [8]. Under the first-level of prevention and control response, schools and classes were suspended and all public places were almost closed. Without outdoor activities and campus life, the injury probability of minors living at home is significantly reduced, so the number of emergency and surgery are significantly reduced. Moreover, the severity of the disease in minors undergoing surgery was lighter than that in the previous two years. Under the response, the direct care of parents (most parents stopped working under the first-level response) can prevent minors from orthopedic trauma, and even if injured, the severity of the injury may be less than that after school beginning. But we still can’t get the conclusion whether the reason for the decrease in minor injuries is the direct care of parents or the prevention and control response. In further research, we will ask the caregiver at the time of the injury in detail, hoping to get more accurate conclusions.

Interestingly, starting with the response, this decrease in the number and severity of injuries is not only reflected in trauma, but also in the number of subluxations of the radial head. The incidence rate of radial head subluxation in girls is higher than that in boys because girls are more likely to be pulled by their parents [9]. Similarly, we can speculate that outdoor activities and campus life will increase the probability of guardians pulling children’s arms. Therefore, parents and preschool teachers should pay more attention to the correct posture when protecting their children in outdoor activities, and avoid direct arm pulling.

After the response level of prevention and control was adjusted to second-level, Shanghai began to recover gradually. But, most of the minors are still staying at home. In the late stage of second-level response, older students from the graduating class returned to the campus (on April 27, 2020, the ninth grade and senior three in Shanghai was resumed). At this time, we found that the number of orthopedic emergency and surgery increased slightly, and the severity of surgical children has begun to be the same as that in previous years. The increase of trauma is directly related to the return of guardians to work and the reduction of family care for young children, which reflects the importance of parents for children’s family care.

Under the third-level response, public places and stadiums have been reopened in Shanghai, and non-graduating students are recommended to the campus. At this stage, the number of orthopedic emergency and surgical operations this year are the same as those in previous years. The severity of the damage has also returned to the level of previous years. We can infer from the data of pediatric orthopedic trauma that most of the minors’ study and life have recovered under the third-level response in Shanghai. It should be noted that the number of cases undergoing surgical treatment due to falling from height has increased compared with previous years. We compared the opening date of school and the date of falling building in recent three years and found that the visiting time of minors with high falling injury was concentrated in the middle of the semester. For example, although the age and school start date of the 10 high falling patients in 2020 are different, they are all injured in the middle of the semester after the beginning of school in their grade (Table 9). We can infer that the pressure on students caused by academic pressure will gradually increase with the progress of the semester. This provides guidance for the future school targeted psychological counseling.

**Table 9:** Information about patients with high falling injure in 2020

Patient No.	Gender	Age(y)	Opening Date (mm/dd)	Injury Date (mm/dd)	diagnose
1	male	10	last term	01/07	fracture of femur
2	male	14	last term	01/13	multiple injuries
3	male	3	–	02/06	Fracture of upper arm
4	female	10	06/02	03/07	fracture of tibia and fibula
5	male	16	04/02	05/07	fracture of spine
6	female	16	04/27	05/10	fracture of pelvis
7	male	17	05/06	05/16	multiple injuries
8	male	15	04/27	05/18	fracture of femur (bilateral)
9	male	14	05/06	05/20	multiple injuries
10	female	13	05/18	06/07	fracture of femur

When we study the impact of prevention and control response on minors’ orthopedic trauma in the pandemic situation, we are bound to be disturbed by the pandemic itself. The city of this study is Shanghai, which was less affected by the pandemic. During the period of study, there was no community-transmitted COVID-19 in Shanghai, and no area to be isolated. Public transportation has not been suspended, and hospital emergency departments have not been closed. Therefore, Shanghai may be one of the most ideal cities to study the impact of China’s prevention and control response on citizens. However, the collection of data in this article still has limitations: we did not collect the injury status of non-surgical patients with mild injuries, which makes this article unable to fully reflect the state of minors’ orthopedic trauma. The collection of the status at the time of injury only included the caregiver and the location. There would be many other factors that can affect the occurrence of the injury under the response, and these uncollected factors may have caused noise to our conclusions. With another outbreak of the pandemic, our research will continue, and we will collect more data to draw more precise conclusions.

## Conclusion

The prevention and control response for the pandemic of COVID-19 can reduce the incidence of orthopedic trauma in minors by strengthening the guardian’s care and restricting children’s outdoor activities. We suggest that parental care may reduce the chance of minors suffering from orthopedic trauma and reduce the severity of trauma. In the middle of the semester, special attention should be paid to the mental health of minors. With the control of the pandemic, the amount of orthopedic trauma in children will not be affected by low-level prevention and control.

## Data Availability

Data supporting the conclusions and outcomes of this article are included in the article. The raw datasets presented and analyzed in this study are available upon request from the corresponding author.

## Abbreviation

COVID: Corona Virus Disease
